# Effects of virtual body-representation on motor skill learning

**DOI:** 10.1038/s41598-022-19514-9

**Published:** 2022-09-10

**Authors:** Yongmin Shin, Jaeseo Lim, Yonggwan Kim, Deog-Gyu Seo, Jungjoon Ihm

**Affiliations:** 1 Dental Research Institute, School of Dentistry, Seoul National University, Seoul, Republic of Korea; 2 Interdisciplinary Program in Cognitive Science, Seoul National University, Seoul, Republic of Korea; 3 Department of Conservative Dentistry, School of Dentistry, Seoul National University, Seoul, Republic of Korea

**Keywords:** Health care, Engineering

## Abstract

Motor learning is often hindered or facilitated by visual information from one’s body and its movement. However, it is unclear whether visual representation of the body itself facilitates motor learning. Thus, we tested the effects of virtual body-representation on motor learning through a virtual reality rotary pursuit task. In the task, visual feedback on participants’ movements was identical, but virtual body-representation differed by dividing the experimental conditions into three conditions: non-avatar, non-hand avatar, and hand-shaped avatar. We measured the differences in the rate of motor learning, body-ownership, and sense of agency in the three conditions. Although there were no differences in body-ownership and sense of agency between the conditions, the hand-shaped avatar condition was significantly superior to the other conditions in the rate of learning. These findings suggest that visually recognizing one’s body shape facilitates motor learning.

## Introduction

Visual feedback on one’s own movement affects motor performance and motor skill learning^1–3^. People typically rely heavily on visual information from both their bodies and the external environment when it comes to motor skill learning and performance. In addition, they utilize visual information to correct and enhance their performance^4^. Recent studies employing virtual reality (VR), have verified that the manipulation of visual feedback (i.e., virtual body-representation) on physical movement can either facilitate or hinder motor learning. For instance, motor learning can be facilitated if the virtual body-representation is congruent with one’s hand laterality, size, or movement, whereas if incongruent, learning can be hindered^5–7^. These results imply the significance of visual perception of the body in motor learning.

Visually recognizing the body affects both motor learning and hippocampus-based episodic memory. When people encode their episodic memories from real-life events, they co-perceive their body and event-related stimulus from a first-person perspective. Several episodic memory studies using VR have found out that the natural perception of people can have effects on both memory registration and recollection. For example, the event-related episodic memory is impaired when people experience life events from a third-person—rather than first-person—perspective^8^. Moreover, even if the event is experienced from a first-person perspective, episodic encoding and retrieval are also impaired if their body is not visually presented in the VR scene or a control object, which replaces their body, is presented^9–11^.

The effect of body perception on such episodic memory is related to bodily self-consciousness. Bodily self-consciousness (the sense of bodily self) derives from the integration of multisensory signals (visual, somatosensory, or motor inputs, etc.) in events. The multisensory integration has an effect on the encoding and recall of episodic memory^10^. The consistent perception of bodily self-consciousness (e.g., experiencing life events from a first perspective) increases the self-relevance of the experienced event, which helps to integrate multisensory information in the event into a unified memory. Therefore, a reduction in bodily self-consciousness (e.g., experiencing life events from a third-person perspective) increases difficulty in the integration, which subsequently hinders episodic retrieval of event details^12^. This relationship between episodic memory and bodily self-consciousness has also been demonstrated by neuroimaging studies. These studies show that bodily self-consciousness exerts influences on brain mechanisms which are responsible for episodic memory formation. Bergouignan et al.^8^ found that repeated recall of memories encoded from a third-person perspective was related to decreased activation of posterior hippocampus compared to that of a first-person perspective. More recently, a study by Gauthier and his colleagues^10^ demonstrated that seeing one’s own body during encoding modulates the functional connectivity between the right hippocampal formation, neocortical regions participated in processing multisensory bodily signals and self-consciousness.

Bodily self-consciousness involves two central components: body-ownership (a feeling that a body part is part of oneself) and sense of agency (a feeling that one initiates and controls one’s volitional actions)^13,14^. Under VR environment, people can feel body-ownership and sense of agency when they perceive congruency of bodily signals with their movements. Specifically, they feel increased body-ownership when the form of virtual avatar is hand-shaped; they do not feel body-ownership when it is in a form of control object (e.g., sphere, rectangle)^15–17^. Furthermore, they feel the optimal sense of agency when predicted sensory feedback and actual sensory feedback like proprioception^18^ match. From this point of view, it can be conjectured that bodily self-consciousness is affected according to the differences in visual perception of one’s own body during motor learning in the VR environment. Given the association between bodily self-consciousness and episodic memory, the variation of bodily self-consciousness may affect memory encoding and recall during the motor learning. Indeed, episodic memory is recruited in the initial stages of motor learning^19,20^. Recent evidence has shown that hippocampus reactivates experienced information including episodic memory during rest periods of early motor learning, which promote rapid improvements in performance^21–23^. Therefore, we expect that body perception in VR scene influences the early learning stage of motor learning and predict that virtual body-representation which resembles a real human body will improve motor learning.

Nevertheless, in previous studies^5–7^, the relationship between visual feedback and motor skill learning was examined by manipulating the existence, size, and congruence of actual movements and visual feedback. Although these studies have suggested the importance of visual feedback according to movement, it is difficult to confirm how the body shape itself affects motor memory and learning. Furthermore, to the best of our knowledge, no studies have investigated the relationship between bodily self-consciousness and motor learning yet by manipulating virtual body-representation, so the impact of bodily self-consciousness on motor learning remains an open issue. Some studies have dealt with the relationship between bodily self-consciousness and motor performance and functioning in VR environment. A study by Seinfeld and colleagues^24^ found that participants who performed the motor task through a Leap motion sensor, seeing one’s virtual hand, felt a stronger sense of body-ownership and the sense of agency and showed better task performance than those who performed the task with a keyboard and could not see one’s avatar. In relation to clinical population, Tambone and colleagues^25^ demonstrated that observing the virtual body’s movements from a first-person perspective helps increase body-ownership, which subsequently promotes stroke patients' motor recovery by accessing their motor functioning. These studies seem to support a positive functional link between virtual embodiment and improved motor abilities and performance. These findings also may suggest there is a possibility that bodily self-consciousness has a positive effect on the improvement of motor performance by repeated practice.

Thus, the purpose of our study is to examine the effect of virtual body-representation on motor learning and the relationship between the motor learning and body-ownership and the sense of agency. In this study, a new task was developed to confirm the effect of body perception on motor skill learning. A VR version of the rotary pursuit task was developed to measure motor skill learning. Traditional rotary pursuit tasks evaluate motor skill learning and hand–eye coordination by determining how much the contact time between a wand and a constant-speed rotating spot target increases with repeated practice^26–28^. In this VR task, all participants can visually check the virtual wand. However, by dividing the experimental conditions into three conditions in which avatars do not appear (i.e., non-avatar condition), avatars do appear but in control objects (i.e., non-hand avatar condition), and body-shaped avatars appear (i.e., hand-shaped avatar condition), visual feedback on movements is identical but virtual body-representation is different.

We predict body-ownership to be the greatest in the hand-shaped avatar condition. We also expect the sense of agency, which is affected by the comparison between predicted and actual sensory feedback^18^, to be identical in all conditions because the movement required in our task is identical in all conditions. Furthermore, we expect that motor skill learning will be greater in the hand-shaped avatar condition than the other two conditions from the early stage of the learning since body-ownership has an impact on episodic memory.

## Method

### Participants

Sixty-three Korean (33 female; *M*_age_ = 23.21, *SD*_age_ = 4.16, *M*_years of education_ = 13.91, *SD*_years of education_ = 2.45) participated in this study. Prior to conducting the VR task, participants rated their handedness using the Edinburgh Handedness Inventory^29^ to adjust the position of the task objects. There were three left-handed participants and each was randomly assigned 21 each to one of three experimental conditions. Among the groups, there were no differences for age (*F* (2, 60) = 0.384, *P* = 0.683), gender (*χ*^2^ (2, *N* = 63) = 0.382, *P* = 0.826), and years of education (*F* (2, 60) = 0.374, *P* = 0.690). The study was conducted in accordance with relevant standards and ethical approval was obtained from the Ethics Committee School of Dentistry Seoul National University (S-D20200045). All participants have completed informed consent to take part in the study.

### VR rotary pursuit task

**Figure 1:**
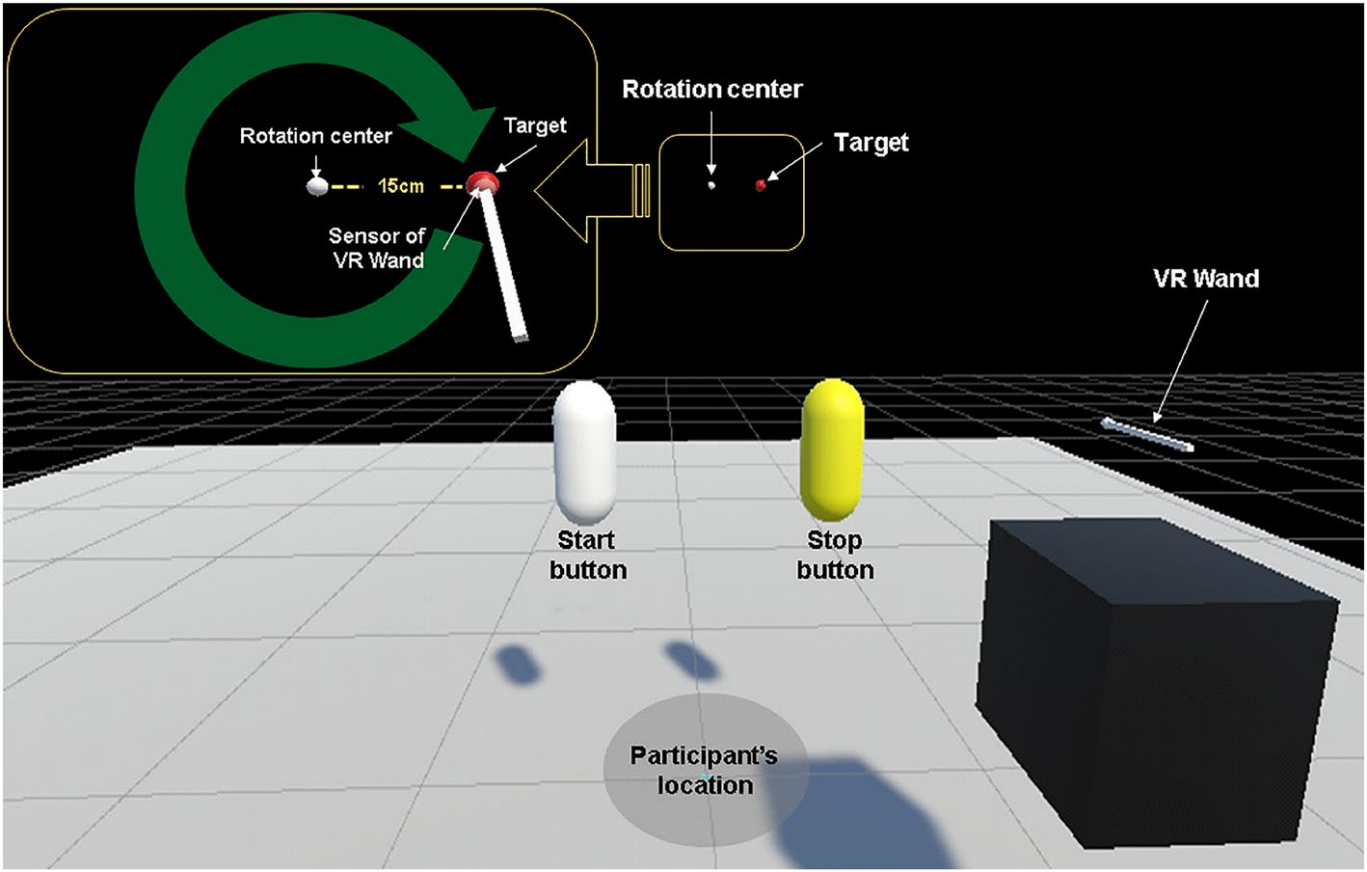
VR rotary pursuit (VRRP) task. In the VRRP task, after touching a start button and holding the VR wand, participants were required to manipulate the controller so that the VR wand sensor contacted a red target moving in a clockwise direction with a radius of 15 cm at a constant speed.

The VR rotary pursuit task (VRRP), which is based on previously developed programs^30,31^, was designed and implemented using Unity v2019.3.5 (see Fig. 1). In the task, a red ball with a radius of 1.5 cm (i.e., target) moved in a clockwise direction with a radius of 15 cm at a constant speed. The participants were shown a VR wand with a round sensor with a radius of 1.0 cm attached to the end and two capsule-shaped buttons (the white one is a start button, and the yellow one is a stop button). After 3 s of sound effects preceding the touching of the start button, holding the VR wand, participants were required to manipulate the controller so that the sensor contacted the target moving in a circular motion for as long as possible. The target was rendered translucent so that the sensor’s contact with the target could be clearly checked. The cumulative time that the sensor contacted the target in each trial was recorded. The recorded time over the entire trial was used for the procedural memory assessment.

### Questionnaire on body-ownership and sense of agency

A questionnaire on body-ownership and sense of agency was completed by the participants following the completion of the VRRP. The questionnaire consisted of nine items selected from the work of Gonzalez-Franco and Peck^32^: two items were about the body-ownership component and eight items were about the agency and motor control component (see Supplementary Table S1). In this study, participants manipulated the VR wand viewing their virtual avatar, which differed depending on their experimental conditions (i.e., hand-shaped avatar, non-hand avatar, and non-avatar). Therefore, the agency and motor control component consisted of two parts, one for the VR wand and the other for the virtual avatar. Participants in the non-avatar condition did not rate body-ownership due to their inability to view their avatar. Each item was rated on a 7-point Likert scale ranging from -3 (strongly disagree) to + 3 (strongly agree).

## Procedure

**Figure 2:**
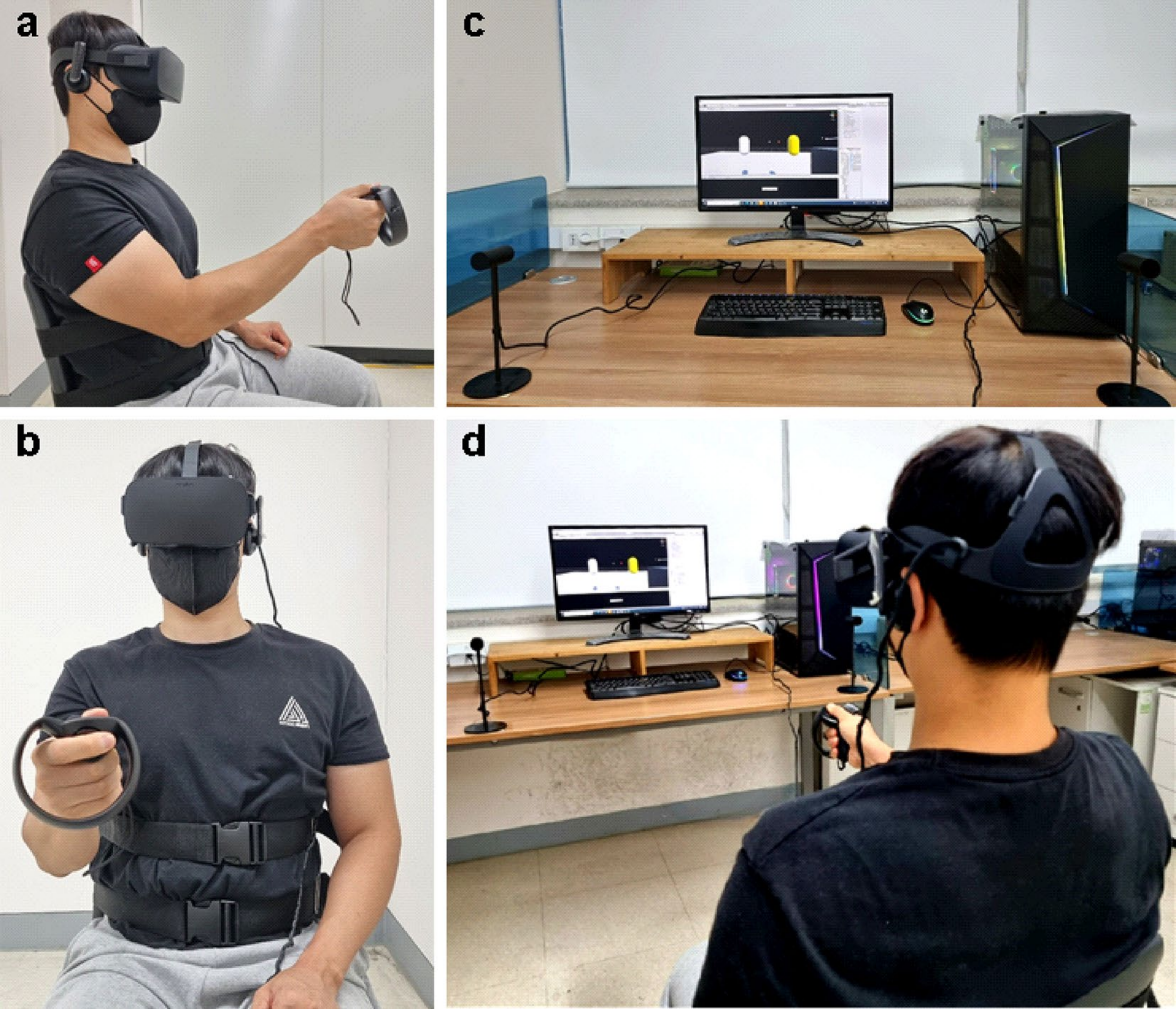
Experimental setup. (**a**) Side view of a participant seating on a fixed chair and wearing belts to fix their upper body positions. (**b**) Front view of the participant. (**c**) Experimental setup for VRRP task. (**d**) Back view of a participant performing VRRP task.

**Figure 3:**
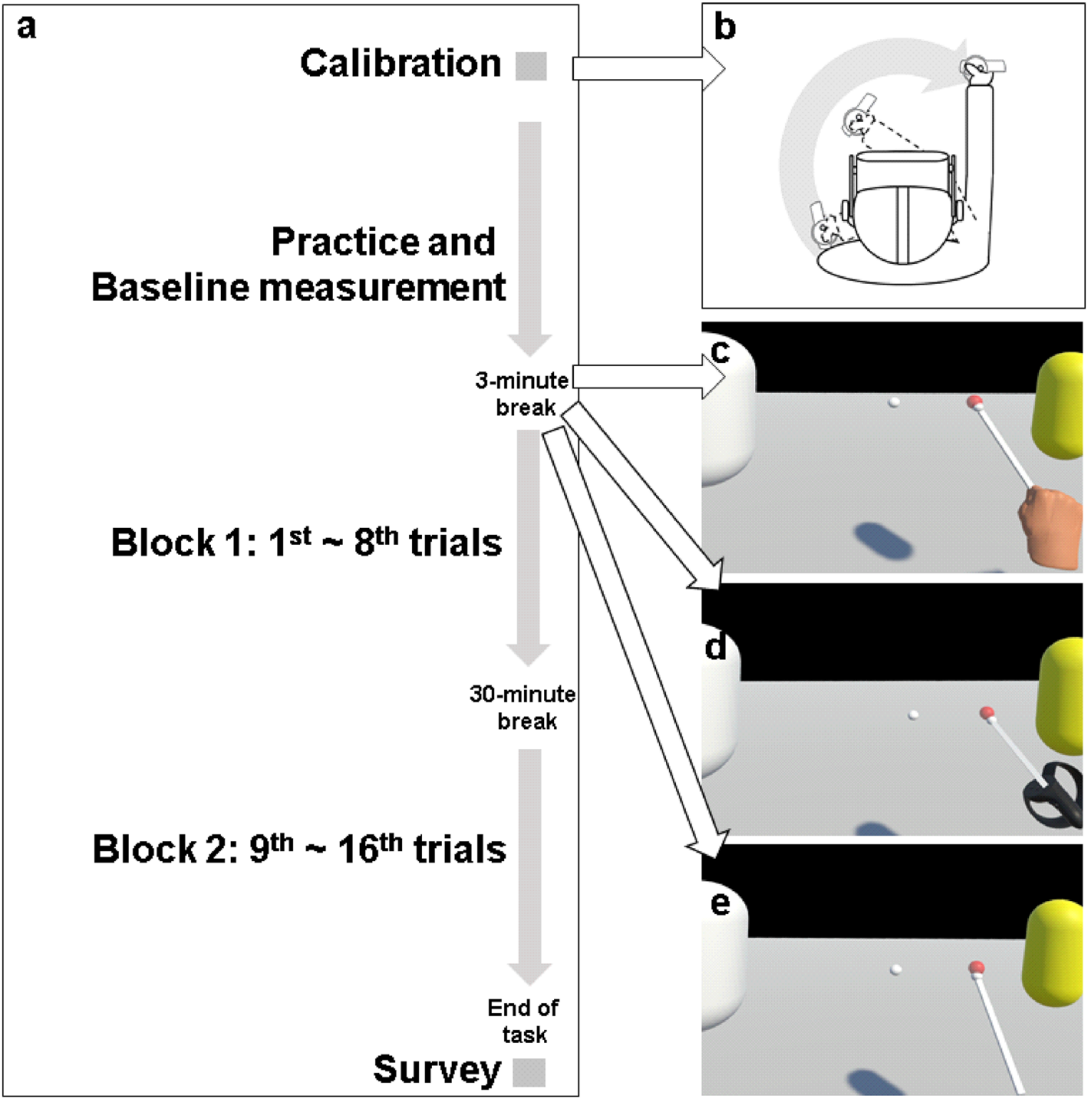
Experimental procedure and conditions. (**a**) Experimental procedure: after calibrating the positioning the target, considering each participant’s shoulder height and arm length, the participants conducted three practice trials—a baseline measurement trial, and sixteen main trials. During a 3-min break after the baseline measurement, participants could see and adapt to their changed avatar according to their assigned experimental conditions. To measure the retention of procedural memory, eight trials were followed by a 30-min break before the remaining eight trials were conducted. At the conclusion of the VRRP task, participants completed a questionnaire on body-ownership and sense of agency. (**b**) The movement for the calibration. (**c**) Experimental condition: hand-shaped avatar. (**d**) Experimental condition: control object-shaped avatar. (**e**) Experimental condition: non-avatar.

For our experiment, a computer with the following specifications was used: Intel i7-9700 K CPU, 16 GB of RAM, and NVIDIA GeForce RTX2080 GPU. Participants wrote a consent form at the laboratory. Subsequently, participants were then seated on a fixed chair and wore belts to fix their upper body positions (see Fig. 2). Wearing a head-mounted display (HMD) Oculus Rift (resolution: 1080 × 1200 pixels at 90 Hz), participants were asked whether 3D objects in the VRRP could be accurately perceived. If the objects were unclear, the inter-pupillary distance for each participant was adjusted using the manual control on the HMD. When participants held the controller with their dominant hand, they were unable to view their action because the hand avatar was rendered invisible in order to measure each individual’s baseline performance in the absence of the avatar effect. However, not only was the VR wand placed close to the dominant hand, but the area around the wand was highlighted when the transparent avatar touched it, making it easier for the subjects to hold. The participants holding the VR wand performed a calibration process to position the target considering their shoulder height and arm length. This process allows them to perform the task under a normalized condition. For this process, participants were required to raise the controller to their shoulder height. In cases where the height of the controller differed from the height of the shoulder, participants bent their arm holding the controller to the opposite shoulder and, stretched it forward again (see Fig. 3b). The target was located near the sensor of the VR wand through the following position formula:

$$P_{x} = S_{x}$$

$$P_{y} = S_{y} \times 0.{95}$$

$$P_{z} = S_{z} \times 0.{8}$$

where *P*_*x*_*, P*_*y*_*,* and *P*_*z*_ are the coordinates for the x-axis [m], y-axis[m], and z-axis[m] of the target when participants stretched their arm forward holding the VR wand, and *S*_*x*_, *S*_*y*_, and *S*_*z*_ are the coordinates of the sensor of the VR wand. In the formula, the two constants in *P*_*y*_ and *P*_*z*_ were determined by a preliminary test for participants’ comfortable movement. Following the calibration, participants were trained on how to contact the tip of the VR wand and track the target moving circularly at a specific speed. Specifically, participants were required to contact the sensor of the VR wand to the target being in a still state in order to become familiar with the movements necessary to perform the task. Then, participants were told that they had to turn their arm holding the VR wand so that the sensor could contact the target moved in a clockwise direction for as long as possible.

In this study, an adaptation of the protocol described by Heindel and colleagues^33^ was employed (see Fig. 3a). Accordingly, participants performed three practice trials, a baseline measurement trial, and sixteen main trials, with a 10-s interval break. In the practice trials, the target moved in a circular orbit at three different speeds (i.e., 15, 30, and 45 rpm) every trial. Before a first practice trial, participants were told that “If you touch the start button with the VR wand, you will hear a beep for 3 s, and in the meantime, you should keep the sensor of the VR wand contacting the target. When the target starts to turn, you can turn the arm holding the VR wand so that the sensor of the VR wand can continue to contact the target.” After brief instruction, the practice trials were conducted. Consecutively, a baseline measurement trial was performed where it rotated at 60 rpm. During three minutes break after those trials, all participants placed the VR wand on the black box (see Fig. 1) and sat still. According to their assigned experimental conditions (see Fig. 3c–e), participants in the hand-shaped avatar condition and the non-hand avatar condition were allowed to view the changed avatar that matched their condition from this break, whereas participants in the non-avatar condition continued to rest while sitting still. In order to adapt to their avatar, the participants who could see their avatar were instructed to sequentially perform opening and closing their hands, rotating their arms, holding the VR wand, and rotating their arms while holding the VR wand again. Afterward, the participants were asked to repeat the motions mentioned above until they fully felt adapted to their avatars. When the participants felt adapted to their avatar, they put the VR wand back on the box and rested for the rest of the time. The main trials were performed for 20 secs each. In addition, to measure the retention of procedural memory, eight trials were followed by a 30-min break before the remaining eight trials were conducted.

## Statistical analysis

We examined the group differences in performances on the VRRP across all the trials (i.e., learning) and the retention of the acquired motor skill at 30 min break intervals (i.e., memory) using a linear mixed-model (LMM) analysis with the *lme4 and nlme* packages in R. In this study, the performances refer to cumulative time measured in seconds that the sensor contacts the target in each 20 s trial. The increased cumulative time denotes better performance. Prior to the main analysis, we conducted a one-way analysis of covariance (ANCOVA) adjusted for demographic variables (i.e., age, gender, and years of education) to investigate the group differences at baseline performance, which may affect the learning on the VRRP. In an analysis of the learning curve (model 1), the trials, the avatar conditions, and the interaction of both were entered into a statistical model as the fixed effect, including the demographic variables as covariates. The 17 trials, including the baseline measurement trial as a categorical variable, were coded to represent sixteen dummy variables. The main effects of the avatar condition were calculated by using reverse helmert contrasts. In the first contrast, the non-avatar condition (0.5) and non-hand avatar condition (− 0.5) were compared. In the second contrast, the hand-shaped avatar condition (− 0.67) was compared to the other conditions (+ 0.33). The regression coefficients of the interaction terms reflect the avatar effects on learning across all the trials. In addition, the random effect structure included a random intercept for the subject and a random time slope for the subject by using time as a continuous variable. For further analysis on procedural memory retention (model 2), we performed the same analysis but modified the trial variable consisting of the ninth and tenth trials and excluded the random time slope from the random effect structure due to the convergence problem. Using the performance package^34^, the models were validated by checking the outliers, linearity, multicollinearity, homoscedasticity, independence of residuals, and the normality of residuals, and marginal and conditional R-squared values were calculated. Even though all assumptions for the LMM was satisfied in the model 1, autocorrelated residuals were detected in the model 2. Therefore, we incorporated an AR (1) covariance structure into the model 2 using nlme package.

In addition, the group difference in body-ownership and sense of agency was measured using a one-way ANCOVA controlling for the demographic variables to confirm the participants’ perception of their VR avatars and VR wand.

## Results

### The rate of motor skill learning

**Figure 4:**
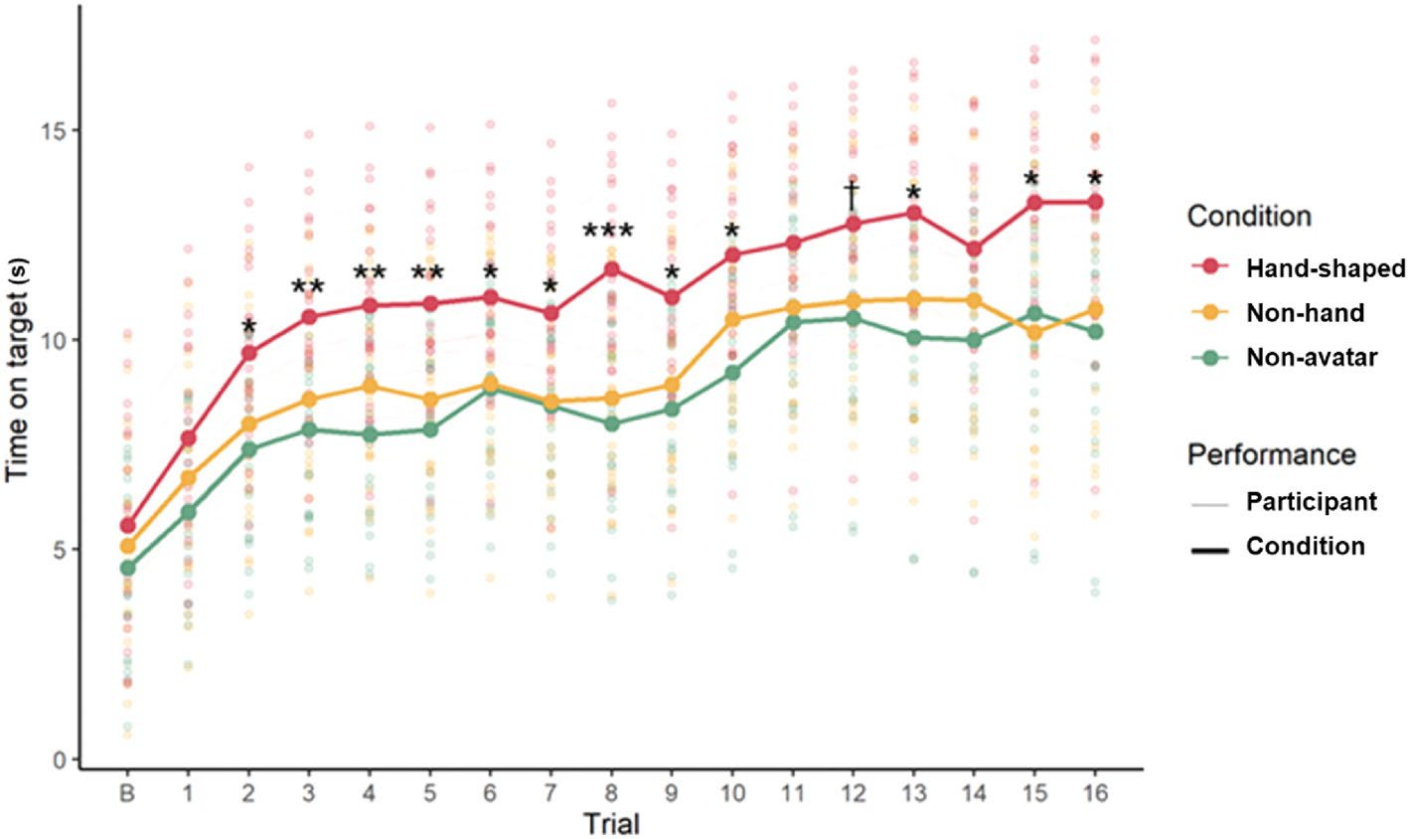
Group differences in the rate of learning. The rate of learning between the non-avatar group (yellow line) and the control object-shaped group (green line) is comparable, but the rate of learning of the hand-shaped avatar group (red line) is significantly superior to that of the other groups across the majority of trials. The letter “B” on the x-axis (Trial) of the graph indicates the baseline measurement. The dark line indicates the rate of learning for each condition, and the light line indicates that for each participant. The asterisks and dagger indicate significance in performance difference for each trial between the hand-shaped group and the other groups, ^†^*p* < 0.1, *p < 0.05, **p < 0.01, ***p < 0.001.

There were no differences in baseline performance among the groups (*F* (2, 57) = 0.615, *P* = 0.544). In our model, regardless of the avatar condition, the performance on the VRRP showed a steadily significant increase across all the trials (see Supplementary Table S2 and S4). An interaction effect of trial and the avatar conditions on the rate of learning was significant although the main effects of the first contrast coefficient (*β* = 0.133, S.E. = 0.794, *P* = 0.868) and the second contrast (*β* = 0.468, S.E. = 0.664, *P* = 0.482) were not. As predicted, there was a comparable rate of learning between the non-avatar group and the non-hand avatar group. However, that of the hand-shaped avatar group was significantly superior to that of the other groups across all the trials except for the first, eleventh, twelfth, and fourteenth trials (see Fig. 4 and Supplementary Table S4).

### The retention of motor skill learning

A second contrast effect (*β* = 2.624, S.E. = 0.651, *P* = 0.0002) was significant, with the hand-shaped avatar group displaying a greater performance of the task than the other groups across the trials. However, there were no significant trial effects and group-by-trial interaction effects, reflecting that the performance was not significantly diminished across trials (*β* = − 0.0004, S.E. = 0.353, *P* = 0.999), with a retention of learning among the groups (first contrast: *β* = 0.023, S.E. = 0.865, *P* = 0.978; second contrast: *β* = − 0.992, S.E. = 0.749, *P* = 0.190).

### Body-ownership and sense of agency

There were no significant differences in the total score from the body-ownership questionnaire between the hand-shaped avatar group and non-hand avatar group (*F* (1, 37) = 0.570, *P* = 0.455) as well as that from the sense of agency questionnaire on the VR wand (*F* (2, 57) = 0.079, *P* = 0.924) and the avatar (*F* (1, 37) = 0.757, *P* = 0.390) among the groups (see Supplementary Table S3).

## Discussion

This study investigated the effects of the visual appearance of the virtual avatar on motor memory when repeating a specific movement using a VR tool. In the VRRP, the presented virtual avatar was visually manipulated depending on the experimental conditions in which participants tracked the target in a circular motion using the VR wand. In each condition, we measured not only the performances across all trials and the retention of the motor skills but also body ownership and sense of agency by the questionnaire at the end of the experiment. In addition, we analyzed the group differences of the obtained measures.

In this study, all of the groups were able to see the movement of the VR wand they had manipulated. Also, the hand-shaped avatar group and the non-hand avatar group could see movements of their avatars. Nevertheless, our findings indicated significant differences in the improvement of the VRRP task performance for each experimental group. The hand-shaped avatar group showed the greatest increase in performance, and the non-hand avatar group showed little difference in performance compared to the non-avatar group. These results suggest that even if participants perceive the same movement, motor skill learning can be improved when viewing bodily visual feedback. This may derive from the unique advantages of body shape.

According to previous research, virtual body-representation is influential in spatial perception in that one’s own body can be used as a familiar cue to scale the size and distance of objects^35–37^. For example, the egocentric distance to the target point is more accurately judged when virtual body-representation is presented than when it is not^36^. In addition, the perception of object sizes^38,39^, as well as the distance judgment^40,41^, are influenced by the size or shape of the virtual body. These findings indicate that virtual body-representation facilitates the judgment of the distance and size of the target.

In our task, motor learning and memory were measured through the amount of time the virtual wand contacted the target. Therefore, it was important for participants to accurately estimate the distance and size of the target. It may be more advantageous for the hand-shaped avatar group to contact the VR wand to the target than the other groups since the more similar the virtual avatar was to one's own body, the greater the accuracy of the judgment on the distance and size of objects^39,41^.

However, the hand-shaped avatar group did not outperform the other groups in all trials. The performance gains of the hand-shaped avatar group gradually increased more than that of the other groups following the second trial. If spatial perception by virtual body-representation influenced the conduction of our task, there should have been a significant difference in performance between the groups in the first trial. Considering that memory consolidation is facilitated during a brief rest period after learning^42^, these results may infer that virtual body-representation impacted the learning rate more than spatial perception in our task.

Several studies have manipulated visual feedback accompanied by physical movement because this feedback is the most influential and effective sensory input in motor performance and learning^1,2,43,44^. Ossmy and Mukamel^6^ found that when participants trained a finger tapping sequence task in a VR environment, performance gains were greatest in the condition where the size of visual feedback (i.e., hand-shaped avatar) was similar to one’s real hand size. Another study demonstrated that visual feedback congruent with one's hand movement increases performance gains the most, while visual feedback incongruent with the movement interferes with performance gains more than when visual feedback was absent^7^. These results indicate that the virtual body-representation facilitates or interferes with motor skill learning depending on the degree to which the feedback matches one's body and actual movement.

Similarly, our findings also emphasize the role of virtual body-representation in motor skill learning. However, they differ from the previous studies in that they proved that the difference in visual representation of the body itself can facilitate or interfere with the learning even when visual stimuli are congruent with the movement of the tool, where one’s body or both is presented. Moreover, our prediction was confirmed, as the results suggest that virtual body-representation affects motor skill learning from the early learning stage. In the initial stage of motor skill training, motor memory acquisition is due to the explicit process in which motor memories sharply improve but also decay rapidly over time^19,45^. In this stage, high-level cognitive strategies and knowledge may be required to plan and adjust one’s movement in response to performance errors, and thus cognitive resources such as attention^46^, executive function^47^., working memory^48^, and episodic memory^49^ were required for this process. In the later stage of the training, implicit learning becomes more dominant than explicit learning by automatizing explicit strategies and knowledge through practice^50^.

We speculated that virtual body-representation facilitates explicit learning. Recent studies have indicated that one’s body or body-shaped avatar representation in a VR environment facilitates episodic memory encoding and retrieval rather than a non-body-shaped avatar or the absence of a virtual body^9–11,51^. That is why episodic encoding requires the co-perception of one’s body and the world in first-person perspective, and the violation of this condition impairs episodic recall^8,9^. These findings are supported by the fact that the body view in the VR increases the intrinsic medial temporal connectivity related to episodic memory performance and modulates the neural substrates of autonoetic consciousness, which allows one to mentally place oneself in the time where a specific event occurred and recall the event^9,52,53^. Given recent findings that rapid improvements in performance occur in the early motor learning stage through hippocampal replay, which is the reactivation of experienced information encompassing episodic memory during waking rest interleaved with practice^21–23^, the significant differences in the performance gain between the groups may result from the effect of virtual body-representation facilitating episodic memory encoding and retrieval.

We predicted that the effect of virtual body-representation derived from bodily self-consciousness. However, there was no difference in the body-ownership and sense of agency questionnaire scores between all of the groups. This result is inconsistent with previous studies which showed that the sense of body-ownership increases more if human body-shaped avatar is presented compared to non-human body-shaped avatar^15–17^. It may be possible to embody one’s avatar even if the realism of the virtual body-representation is violated because simultaneous and congruent visuo-motor combination with the avatar strongly influences the embodiment^54,55^. In addition, there is another possibility that the discrepancies in body-ownership between the three groups that existed at the initial embodiment phase(i.e., 3-min break) may have disappeared after completing the task. This possibility is supported by the fact that the decreased bodily self-consciousness can be recovered as motor learning progressed^14^. Therefore, it may have been necessary to measure body-ownership and sense of agency both at the initial embodiment phase and after completing the task. Or, rather than the measurement of subjective embodiment, other measurements like autonoetic consciousness or experiential ownership^56^ which is the sense of experiencing the current event for oneself might better explain the relationship between the subjective experience and performance gain.

On the other hand, the absence of the discrepancy in body-ownership can raise the possibility of an alternative explanation for our findings. One possibility is that the hand shape itself may impact participants’ attention to the task. Previous studies found that people have attentional prioritization in space proximal to their hand, so they make faster responses to objects near their hands than objects far from their hands^57,58^. In this light of view, although virtual avatars were presented in our study, attention to the VR wand or the target may have increased under the hand-shaped avatar condition compared to other conditions. Since attentional resources are recruited during early stages of motor learning and decrease in resources can hamper motor learning, the learning rate of the hand-shaped avatar condition may be better than that of other conditions^46^.

Our study has the following limitations. The effect of the hand-shaped avatar was weakened in the second block of the task. Consequently, it is surmised that the avatar has no effect when long-term learning is undertaken. However, in our task, it was difficult to grasp the change in the long-term learning pattern due to the limitation of the number of trials. Therefore, future studies should identify the long-term effect of the avatar to clarify that the effect only appears in the early stages of motor skill learning. In addition, it is difficult to test the avatar effect on long-term memory for motor skill learning because participants took a 30-min break in the wakeful state. Both declarative and procedural memory are consolidated through sleep^59^, and it is also required to manipulate post training delays (e.g., one day, one week) to identify the interaction effect between virtual body-representation and sleep on the long-term retention. Finally, we employed a VR task to measure simple motor skill learning and did not reflect the functional and physical characteristics of a real tool but a simple virtual tool. Therefore, it may be easy to generalize our results because a simple VR task manipulating only visual stimuli was used. However, our results are limited to reflect complex motor skill learning using tools that occur in real life. In future studies, it would be necessary to generalize whether the results will be replicated in complex tasks.

In conclusion, by manipulating the visual representation of the self-avatar, we confirmed that the hand-shaped avatar can improve learning speed in tool-based motor skill learning. To the best of our knowledge, this is the first study that demonstrates the effect of virtual body-representation in VR tool-based motor skill learning. Our findings emphasize the importance of virtual body-representation and suggest a more effective framework for training in a VR environment. Motor learning or motor training programs are usually displayed on a two-dimensional screen and people interact with programs through a symbolic virtual representation of their bodies like cursors^60^. Through 2D screens, people cannot view their bodies or experience decreased embodiment. Also, reduced depth cues and eye-hand coordination in 2D screen might add cognitive load to performers^61,62^. Although these limitations exist due to the nature of 2D screens, we believe that virtual body-preservations may solve some of its limitations. In doing so, further research on the application and generalization of virtual body-preservation need to be done. We hope that these findings can be employed as a reference to develop rehabilitation, medical training, and sport training programs for effective skill learning.

## Supplementary Information

Supplementary Information.

## Data Availability

The dataset generated and analyzed during the current study and corresponding syntax are available from the first author on reasonable request.
