# Supplementary material for: Effects of virtual body-representation on motor skill learning
Source: Sci Rep. 2022 Sep 10;12:15283. doi: 10.1038/s41598-022-19514-9 (PMC9464243; doi:10.1038/s41598-022-19514-9)
Supplement: Supplementary file 1 — Supplementary Information. [file 41598_2022_19514_MOESM1_ESM.docx]

**Supplementary information**

Effects of virtual body-representation on motor skill learning

**Table S1**. A questionnaire on body-ownership and sense of agency

| **Body-ownership** |
| --- |
| Q1. I felt as if the virtual avatar was my body.  Q2. It felt as if the virtual avatar I saw was someone else. |
| **Sense of agency and motor control: virtual avatar** |
| Q1. It felt like I could control the virtual avatar as if it was my own body.  Q2. The movements of the virtual avatar were caused by my movements.  Q3. I felt as if the movements of the virtual avatar were influencing my own movements.  Q4. I felt as if the virtual avatar was moving by itself. |
| **Sense of agency and motor control: virtual wand** |
| Q1. It felt like I could control the virtual wand as if it was my own body.  Q2. The movements of the virtual wand were caused by my movements.  Q3. I felt as if the movements of the virtual wand were influencing my own movements.  Q4. I felt as if the virtual wand was moving by itself. |

**Table S2**. Descriptive statistics on VRRP task performance for each condition.

| **Trial** | **Condition** | **Mean** | **Standard deviation** |
| --- | --- | --- | --- |
| baseline | Hand-shaped avatar | 5.564 | 2.429 |
|  | Non-avatar | 5.075 | 2.007 |
|  | Control object-shaped avatar | 4.561 | 1.769 |
| Trial1 | Hand-shaped avatar | 7.655 | 2.657 |
|  | Non-avatar | 6.710 | 2.507 |
|  | Control object-shaped avatar | 5.884 | 2.247 |
| Trial2 | Hand-shaped avatar | 9.689 | 2.898 |
|  | Non-avatar | 7.989 | 3.108 |
|  | Control object-shaped avatar | 7.383 | 2.539 |
| Trial3 | Hand-shaped avatar | 10.546 | 2.916 |
|  | Non-avatar | 8.571 | 3.169 |
|  | Control object-shaped avatar | 7.856 | 1.970 |
| Trial4 | Hand-shaped avatar | 10.812 | 2.644 |
|  | Non-avatar | 8.896 | 2.985 |
|  | Control object-shaped avatar | 7.739 | 2.738 |
| Trial5 | Hand-shaped avatar | 10.865 | 3.090 |
|  | Non-avatar | 8.569 | 3.349 |
|  | Control object-shaped avatar | 7.861 | 2.667 |
| Trial6 | Hand-shaped avatar | 11.020 | 2.747 |
|  | Non-avatar | 8.961 | 2.851 |
|  | Control object-shaped avatar | 8.837 | 3.051 |
| Trial7 | Hand-shaped avatar | 10.635 | 2.656 |
|  | Non-avatar | 8.537 | 2.727 |
|  | Control object-shaped avatar | 8.428 | 2.603 |
| Trial8 | Hand-shaped avatar | 11.681 | 2.491 |
|  | Non-avatar | 8.606 | 3.468 |
|  | Control object-shaped avatar | 8.004 | 3.217 |
| Trial9 | Hand-shaped avatar | 11.019 | 3.382 |
|  | Non-avatar | 8.925 | 2.621 |
|  | Control object-shaped avatar | 8.346 | 2.809 |
| Trial10 | Hand-shaped avatar | 12.018 | 2.925 |
|  | Non-avatar | 10.478 | 2.651 |
|  | Control object-shaped avatar | 9.209 | 2.703 |
| Trial11 | Hand-shaped avatar | 12.305 | 3.233 |
|  | Non-avatar | 10.778 | 2.924 |
|  | Control object-shaped avatar | 10.415 | 3.091 |
| Trial12 | Hand-shaped avatar | 12.767 | 3.073 |
|  | Non-avatar | 10.926 | 2.449 |
|  | Control object-shaped avatar | 10.505 | 2.157 |
| Trial13 | Hand-shaped avatar | 13.030 | 2.711 |
|  | Non-avatar | 10.971 | 3.170 |
|  | Control object-shaped avatar | 10.246 | 2.958 |
| Trial14 | Hand-shaped avatar | 12.177 | 3.223 |
|  | Non-avatar | 10.941 | 3.309 |
|  | Control object-shaped avatar | 10.236 | 3.360 |
| Trial15 | Hand-shaped avatar | 13.265 | 2.615 |
|  | Non-avatar | 10.175 | 3.731 |
|  | Control object-shaped avatar | 10.832 | 3.093 |
| Trial16 | Hand-shaped avatar | 13.286 | 2.485 |
|  | Non-avatar | 10.726 | 3.186 |
|  | Control object-shaped avatar | 10.376 | 3.457 |

**Table S3**. Descriptive statistics on body ownership and sense of agency for each condition.

| **Variables** | **Condition** | **Mean** | **Standard deviation** |
| --- | --- | --- | --- |
| Body ownership | Hand-shaped avatar | 1.714 | 2.552 |
|  | Non-avatar | - | - |
|  | Control object-shaped avatar | 2.048 | 1.910 |
| Sense of agency: Virtual avatar | Hand-shaped avatar | 5.476 | 2.358 |
|  | Non-avatar | - | - |
|  | Control object-shaped avatar | 5.000 | 2.049 |
| Sense of agency: Virtual wand | Hand-shaped avatar | 3.662 | 2.202 |
|  | Non-avatar | 3.952 | 2.376 |
|  | Control object-shaped avatar | 3.238 | 2.488 |

**Linear Mixed-Model (LMM) Syntax**

*lmer(The amount of time ~ Gender+Age+Education+(Reverse helmert contrasts_1 + Reverse helmert contrasts_2)*Trials+(1 +Trials| Participant)*

1. *The amount of time:* The amount of time the virtual wand is in contact with the target.
2. *Reverse helmert contrasts_1:* The first contrast between Non-avatar condition and Control object-shaped avatar condition.
3. *Reverse helmert contrasts_2:* The second contrast between the mean of the first two conditions and Hand-shaped avatar condition.

**Table S4**. LMM analysis of the effect of virtual body-representation on motor skill learning. The marginal and conditional R^2^ for this model was 0.45 and 0.82.

| **Fixed effect** | **Estimate** | **Standard error** | **df** | ***t*** | ***P*** |
| --- | --- | --- | --- | --- | --- |
| (Intercept) | 5.653 | 1.515 | 58.794 | 3.732 | 0.001 |
| Gender | -2.370 | 0.511 | 57.001 | -4.637 | 2.11×10^-5^ |
| Age | -0.019 | 0.108 | 57.001 | -1.786 | 0.079 |
| Education | -0.373 | 0.184 | 57.001 | 2.024 | 0.048 |
| Reverse Helmert contrasts_1 | 0.133 | 0.794 | 104.930 | 0.167 | 0.868 |
| Reverse Helmert contrasts_2 | 0.468 | 0.664 | 108.159 | 0.705 | 0.482 |
| Trials 1 | 1.679 | 0.264 | 912.059 | 6.368 | 3.03×10^-10^ |
| Trials 2 | 3.283 | 0.266 | 940.380 | 12.344 | < 2×10^-16^ |
| Trials 3 | 3.921 | 0.270 | 959.917 | 14.536 | < 2×10^-16^ |
| Trials 4 | 4.079 | 0.275 | 933.722 | 14.837 | < 2×10^-16^ |
| Trials 5 | 4.028 | 0.281 | 840.573 | 14.314 | < 2×10^-16^ |
| Trials 6 | 4.535 | 0.289 | 700.908 | 15.685 | < 2×10^-16^ |
| Trials 7 | 4.129 | 0.298 | 556.568 | 13.854 | < 2×10^-16^ |
| Trials 8 | 4.360 | 0.308 | 434.872 | 14.154 | < 2×10^-16^ |
| Trials 9 | 4.359 | 0.319 | 342.120 | 13.669 | < 2×10^-16^ |
| Trials 10 | 5.498 | 0.331 | 274.208 | 16.625 | < 2×10^-16^ |
| Trials 11 | 6.095 | 0.343 | 224.854 | 17.759 | < 2×10^-16^ |
| Trials 12 | 6.329 | 0.356 | 188.680 | 17.756 | < 2×10^-16^ |
| Trials 13 | 6.282 | 0.370 | 161.756 | 16.964 | < 2×10^-16^ |
| Trials 14 | 5.968 | 0.385 | 141.355 | 15.515 | < 2×10^-16^ |
| Trials 15 | 6.290 | 0.400 | 125.616 | 15.743 | < 2×10^-16^ |
| Trials 16 | 6.329 | 0.415 | 113.264 | 15.255 | < 2×10^-16^ |
| Reverse Helmert contrasts_1:Trials 1 | 0.307 | 0.646 | 912.059 | 0.476 | 0.634 |
| Reverse Helmert contrasts_1:Trials 2 | 0.088 | 0.651 | 940.380 | 0.135 | 0.892 |
| Reverse Helmert contrasts_1:Trials 3 | 0.197 | 0.661 | 959.917 | 0.298 | 0.766 |
| Reverse Helmert contrasts_1:Trials 4 | 0.639 | 0.673 | 933.722 | 0.949 | 0.343 |
| Reverse Helmert contrasts_1:Trials 5 | 0.189 | 0.689 | 840.573 | 0.275 | 0.784 |
| Reverse Helmert contrasts_1:Trials 6 | -0.395 | 0.708 | 700.908 | -0.557 | 0.577 |
| Reverse Helmert contrasts_1:Trials 7 | -0.409 | 0.730 | 556.568 | -0.560 | 0.576 |
| Reverse Helmert contrasts_1:Trials 8 | 0.084 | 0.754 | 434.872 | 0.112 | 0.911 |
| Reverse Helmert contrasts_1:Trials 9 | 0.061 | 0.781 | 342.120 | 0.078 | 0.938 |
| Reverse Helmert contrasts_1:Trials 10 | 0.751 | 0.810 | 274.208 | 0.927 | 0.355 |
| Reverse Helmert contrasts_1:Trials 11 | -0.156 | 0.841 | 224.854 | -0.185 | 0.853 |
| Reverse Helmert contrasts_1:Trials 12 | -0.098 | 0.873 | 188.680 | -0.112 | 0.911 |
| Reverse Helmert contrasts_1:Trials 13 | 0.398 | 0.907 | 161.756 | 0.439 | 0.661 |
| Reverse Helmert contrasts_1:Trials 14 | 0.425 | 0.942 | 141.355 | 0.451 | 0.653 |
| Reverse Helmert contrasts_1:Trials 15 | -0.984 | 0.979 | 125.616 | -1.006 | 0.316 |
| Reverse Helmert contrasts_1:Trials 16 | 0.023 | 1.016 | 113.264 | 0.022 | 0.982 |
| Reverse Helmert contrasts_2:Trials 1 | 0.608 | 0.559 | 912.059 | 1.088 | 0.277 |
| Reverse Helmert contrasts_2:Trials 2 | 1.253 | 0.564 | 940.380 | 2.222 | 0.027 |
| Reverse Helmert contrasts_2:Trials 3 | 1.583 | 0.572 | 959.917 | 2.766 | 0.006 |
| Reverse Helmert contrasts_2:Trials 4 | 1.745 | 0.583 | 933.722 | 2.993 | 0.003 |
| Reverse Helmert contrasts_2:Trials 5 | 1.901 | 0.597 | 840.573 | 3.184 | 0.002 |
| Reverse Helmert contrasts_2:Trials 6 | 1.371 | 0.613 | 700.908 | 2.235 | 0.026 |
| Reverse Helmert contrasts_2:Trials 7 | 1.403 | 0.632 | 556.568 | 2.218 | 0.027 |
| Reverse Helmert contrasts_2:Trials 8 | 2.626 | 0.653 | 434.872 | 4.019 | 0.000 |
| Reverse Helmert contrasts_2:Trials 9 | 1.634 | 0.677 | 342.120 | 2.415 | 0.016 |
| Reverse Helmert contrasts_2:Trials 10 | 1.425 | 0.702 | 274.208 | 2.032 | 0.043 |
| Reverse Helmert contrasts_2:Trials 11 | 0.959 | 0.728 | 224.854 | 1.317 | 0.189 |
| Reverse Helmert contrasts_2:Trials 12 | 1.302 | 0.756 | 188.680 | 1.722 | 0.087 |
| Reverse Helmert contrasts_2:Trials 13 | 1.767 | 0.785 | 161.756 | 2.249 | 0.026 |
| Reverse Helmert contrasts_2:Trials 14 | 0.957 | 0.816 | 141.355 | 1.173 | 0.243 |
| Reverse Helmert contrasts_2:Trials 15 | 2.107 | 0.848 | 125.616 | 2.486 | 0.014 |
| Reverse Helmert contrasts_2:Trials 16 | 2.080 | 0.880 | 113.264 | 2.364 | 0.020 |
